# Diagnosing dementia in the Arctic: translating tools and developing and validating an algorithm for assessment of impaired cognitive function in Greenland Inuit

**DOI:** 10.1192/j.eurpsy.2022.459

**Published:** 2022-09-01

**Authors:** I. Kleist, P. Noahsen, O. Gredal, J. Riis, S. Andersen

**Affiliations:** 1 Aarhus University Hospital, Forensic Psychiatric, Aarhus N, Denmark; 2 eNational Board of Health in Greenland, Enational Board Of Health In Greenland, Nuuk, Greenland; 3 Queen Ingrid’s Hospital, Nuuk, Greenland, Department Of Internal Medicine, Nuuk, Greenland; 4 Aalborg University Hospital, Department Of Geriatric Medicine,, Aalborg, Denmark

**Keywords:** mini-cog, Dementia, RUDAS, cognitive function

## Abstract

**Introduction:**

The ageing Arctic populations raise the need for work-up of cognitive function that reflects language and cultural understandings.

**Objectives:**

To translate and evaluate tools for work-up of cognitive impairment in Greenland.

**Methods:**

Step A: An expert panel was established to select tools suitable for the work-up of cognitive impairment at three different settings in Greenland. Step B: Tools were translated in a multiple-step process of independent translations with back-translation and adaptations by two independent translators and two Greenlandic physicians. Step C: a testing and validation process of the tools at three locations: the national hospital in the capital city; regional hospital in a town; health care centre in a small town.

**Results:**

Tools selected were Mini-Cog and RUDAS. Participants for testing of tools were 43 of 61 invited, of which six had dementia. RUDAS and Mini-Cog scores were associated (p < 0.001). The smoothed AUC was 0.87 (95%-CI, 0.65–0.95) for Mini-Cog and 0.90 (95%-CI, 0.76–0.97) for RUDAS. The sensitivity of Mini-Cog with a cut-off at ≤3 was 83.3%, and specificity was 62.2%. For RUDAS with a cut-off at ≤23, these were 100% and 75.7%, respectively.

**Conclusions:**

Requested tools have been translated for assessing cognitive function in the native Arctic setting. Small town residents with a Mini-Cog score of 3 or lower should be referred to a regional hospital for RUDAS, and a score of 23 or less should cause referral to the national hospital for a full work-up of cognitive function.

**Disclosure:**

No significant relationships.

